# Postoperative diastolic perfusion pressure is associated with the development of acute kidney injury in patients after cardiac surgery: a retrospective analysis

**DOI:** 10.1186/s12882-019-1632-3

**Published:** 2019-12-10

**Authors:** Jifu Jin, Jiawei Yu, Su Chi Chang, Jiarui Xu, Sujuan Xu, Wuhua Jiang, Bo Shen, Yamin Zhuang, Chunsheng Wang, Xiaoqiang Ding, Jie Teng

**Affiliations:** 1 Department of Nephrology, Zhongshan Hospital, Shanghai Medical College, Fudan University, 180 Fenglin Road, Shanghai, 200032 China; 2 Shanghai Medical Center of Kidney Disease, Shanghai, China; 3 Shanghai Institute of Kidney and Dialysis, Shanghai, China; 4 Shanghai Key Laboratory of Kidney and Blood Purification, Shanghai, China; 5 Department of Cardiology, Zhongshan Hospital, Shanghai Medical College, Fudan University, Shanghai, China; 6 Department of Intensive Care Medicine, Zhongshan Hospital, Shanghai Medical College, Fudan University, Shanghai, China; 7 Department of Cardiovascular Surgery, Zhongshan Hospital, Shanghai Medical College, Fudan University, Shanghai, China; 8 Department of Nephrology, Xiamen Branch, Zhongshan Hospital, Fudan University, Xiamen, Fujian China

**Keywords:** Diastolic perfusion pressure, Central venous pressure, Cardiac surgery, Acute kidney injury

## Abstract

**Background:**

We aimed to investigate the relationship between the perioperative hemodynamic parameters and the occurrence of cardiac surgery-associated acute kidney injury.

**Methods:**

A retrospective study was performed in patients who underwent cardiac surgery at a tertiary referral teaching hospital. Acute kidney injury was determined according to the KDIGO criteria. We investigated the association between the perioperative hemodynamic parameters and cardiac surgery-associated acute kidney injury to identify the independent hemodynamic predictors for acute kidney injury. Subgroup analysis was further performed in patients with chronic hypertension.

**Results:**

Among 300 patients, 29.3% developed acute kidney injury during postoperative intensive care unit period. Multivariate logistic analysis showed the postoperative nadir diastolic perfusion pressure, but not mean arterial pressure, central venous pressure and mean perfusion pressure, was independently linked to the development of acute kidney injury after cardiac surgery (odds ratio 0.945, *P* = 0.045). Subgroup analyses in hypertensive subjects (*n* = 91) showed the postoperative nadir diastolic perfusion pressure and peak central venous pressure were both independently related to the development of acute kidney injury (nadir diastolic perfusion pressure, odds ratio 0.886, *P* = 0.033; peak central venous pressure, odds ratio 1.328, *P* = 0.010, respectively).

**Conclusions:**

Postoperative nadir diastolic perfusion pressure was independently associated with the development of cardiac surgery-associated acute kidney injury. Furthermore, central venous pressure should be considered as a potential hemodynamic target for hypertensive patients undergoing cardiac surgery.

## Background

Acute kidney injury (AKI) is a frequent and severe complication after cardiac surgery, which has shown to be related to the increased morbidity, mortality and resource utilization [1–4]. The Acute Kidney Injury-Epidemiologic Prospective Investigation (AKI-EPI) study revealed the incidence of AKI using the complete Kidney Disease: Improving Global Outcomes (KDIGO) criteria in critically ill patients was 57.3% [5, 6]. The cause of AKI is multifactorial and not completely understood yet. Multiple studies have identified risk factors for CSA-AKI that cannot be modified, including age, gender, baseline kidney function, diabetes mellitus and hypertension [6].

However, hemodynamic parameters, as modifiable risk factors, may be related to the progression of AKI in high-risk patients [7–11]. A number of studies have reported that an increased central venous pressure (CVP) is linked to the venous congestion and deterioration of kidney function in patients with cardiovascular disease and sepsis [12–14]. In addition, perfusion pressure – defined as the difference between arterial pressure and CVP – has been shown to increase the risk of developing AKI [15–17].

To date, a paucity of research has extensively studied the potential links between the systemic hemodynamic parameters, including postoperative extreme values (peak and nadir) of CVP, mean perfusion pressure (MPP), systolic perfusion pressure (SPP), diastolic perfusion pressure (DPP), and the development of AKI in cardiac surgery patients.

Accordingly, we investigated potential relationships between profound hemodynamic parameters (including MAP, CVP, DPP, MPP, and SPP) and CSA-AKI to establish if patients with AKI exhibited a lower perfusion pressure and higher CVP compared to patients not suffering from AKI.

## Methods

### Patients

We included all patients admitted in our ICU after cardiac surgery for a period of 3-months (June 1st-Septemper 1st 2015). The Institutional Ethics Committee of Zhongshan Hospital granted permission to study design and data collection, and the need for informed consents were waived because of the noninterventional design of the study.

### Inclusion and exclusion criteria

The inclusion criteria were: adult patients (age ≥ 18 years) who underwent elective cardiac surgery with or without CPB and stayed in the intensive care unit (ICU) for ≥24 h following surgery. Exclusion criteria were: patients who underwent cardiac transplantation or emergency operations; patients who had a history of previous renal replacement therapy or kidney transplantation; patients who were deceased ≤24 h after they were admitted to the ICU.

### Hemodynamic parameters

Baseline blood pressure (BP) were measured at least three times prior to surgery and averaged for estimation of baseline hemodynamic values. Baseline MAP was estimated from diastolic arterial pressure (DAP) and systolic arterial pressure (SAP) using the standard formula: MAP = DAP + (SAP - DAP) / 3.

Intraoperative BP values were measured every 10 min during the surgical period using a radial artery line, which was routinely positioned for hemodynamic monitoring. Time-weighted average (TWA) of intraoperative MAP values during CPB period were estimated as well. Systolic blood pressure values > 299 mmHg or < 25 mmHg were regarded as errors and excluded from the analysis as in the previous study [18].

Differences between the baseline MAP and intraoperative TWA-MAP were presented as absolute delta MAP (baseline MAP – intraoperative TWA-MAP) as well as relative delta MAP (absolute delta MAP/baseline MAP × 100%), respectively.

Postoperative BP values were measured every 15 min for 24 h during the ICU period, and postoperative TWA-MAP was estimated over 24 h during such period. Given that hemodynamic parameters variates during the initial phase of ICU admission, the upper limits of the range (ULR) and lower limits of the range (LLR) over the first 24 h were recorded as well. The ULR of each hemodynamic parameter was defined as the highest value achieved within 24 h whereas the LLR was defined as the lowest value achieved within 24 h after ICU admission. The ULR and LLR of CVP within 24 h after surgery were also measured. Postoperative MPP was then calculated from postoperative TWA-MAP within 24 h and extrema (ULR or LLR) values of CVP (MPP = MAP – CVP). Similarly, postoperative DPP and SPP were calculated from postoperative TWA-DAP or TWA-SAP and extrema (ULR or LLR) values of CVP, respectively (DPP = DAP – CVP; SPP = SAP – CVP; respectively) [17].

### Other variables

An estimate of the glomerular filtration rate (eGFR) was calculated by the Modification of Diet in Renal Disease (MDRD) formula based on the patient characteristics and baseline serum creatinine, which was defined as the latest measurement prior to surgery. Serum creatinine was measured at least once a day during the postoperative ICU period as a standard of care. All of the subjects were separated into two groups depending on whether they suffered from AKI, defined according to the criteria published in the KDIGO guidelines [19].

Demographic data including gender, age, body weight, height, comorbidities and surgery type, surgical variables, vasopressin administration, fluid administration, erythrocytes transfusion, plasma transfusion, estimated intraoperative blood loss, intraoperative and postoperative urine output, mechanical ventilation time, length of ICU stay, and hospitalization time were obtained from the anesthetic database or electronic medical records.

### Statistical analysis

Continuous data were shown as means ± standard deviation or as a median (interquartile range) whereas categorical data were presented as numbers (%). Potential differences between continuous variables were assessed using a *t*-test or Mann-Whitney U-test whereas changes in categorical variables were compared using Fisher’s exact test or chi-squared tests in patients with or without CSA-AKI.

Patient characteristics, intraoperative variables and postoperative variables were compared using the univariable logistic regression analysis to determine the risk factors for CSA-AKI. We calculated the odds ratio (OR) with 95% confidence interval (CI) for the predictors for AKI. The multivariable logistic regression analysis was further performed using the forward stepwise selection for the hemodynamic variables with a *P* value of < 0.05 considered to be predictive for CSA-AKI. Given that CPB duration and cross-clamp time were colinear factors, we enrolled only CPB duration in the multivariable analysis. After adjusting for the confounding factors (age, gender, baseline serum creatinine, baseline left ventricular ejection fraction [LVEF], CPB duration, fluid balance, and positive end-expiratory pressure [PEEP]) for the occurrence of AKI, we established independent hemodynamic predictors for AKI development. Subgroup analyses were further performed in hypertensive subjects to establish the independent hemodynamic risk factors for CSA-AKI. Statistical significance was considered to be a two-tailed *P* value ≤0.05. All of the analyses were conducted using SPSS Statistics for Windows (ver. 18.0, SPSS Inc., US).

## Results

### Basic characteristics

In total, 300 cardiac surgery patients (173 men, mean age 54.5 years) were enrolled in our study. Eighty-eight (29.3%) patients developed CSA-AKI based on the KDIGO criteria (25.0, 2.7 and 1.6% in stages 1 to 3, respectively). Baseline serum creatinine levels were significantly higher in AKI patients when compared to non-AKI patients (85.2 ± 27.0 μmol/L vs. 76.7 ± 19.6 μmol/L; *P* = 0.003). Acute Physiology and Chronic Health Evaluation III (APACHE III) score, EuroSCORE II at ICU admission, and surgery types were comparable in different patient groups. CPB duration as well as cross-clamp time in AKI group was remarkably longer than non-AKI group (108.4 ± 37.1 min vs. 90.1 ± 31.8 min, *P* < 0.05; 66.7 ± 25.6 min vs. 55.8 ± 24.9 min, *P* < 0.05). The length of ICU stay and total hospitalization time were statistically longer in AKI patients (both *P* < 0.05). In-hospital mortality rate was significantly higher in AKI patients (5.7% vs. 1.4%, *P* = 0.037) (Table 1).

**Table 1:** Patient characteristics according to the presence or absence of AKI

	Non-AKI	AKI	*P*-value
Total	212 (70.7)	88 (29.3)	–
Male (%)	102 (48.1)	71 (80.7)	< 0.001
Age (years)	52.9 ± 14.3	58.1 ± 12.1	0.002
BMI (kg/m^2^)	22.9 ± 3.1	24.1 ± 3.7	0.090
Comorbidities			
CKD (%)	10 (4.7)	10 (11.4)	0.036
Hypertension (%)	57 (26.9)	34 (38.6)	0.044
Diabetes (%)	17 (8.0)	11 (12.5)	0.224
Cerebrovascular disease (%)	11 (5.2)	3 (3.4)	0.506
Atrial fibrillation (%)	40 (18.9)	17 (19.3)	0.928
Hyperlipidemia (%)	6 (2.8)	2 (2.3)	0.785
Medication before admission			
Aspirin (%)	31 (14.6)	8 (9.1)	0.195
CCB (%)	14 (6.6)	10 (11.4)	0.166
ACEI (%)	9 (4.2)	1 (1.1)	0.172
ARB (%)	11 (3.7)	4 (1.3)	0.816
Diuretics (%)	6 (2.0)	5 (1.7)	0.231
β-blocker (%)	15 (7.1)	6 (6.8)	0.937
Statins (%)	13 (6.1)	1 (1.1)	0.062
Preoperative contrast exposure(%)	118 (55.7)	51 (58.0)	0.715
Intervals between contrast exposure and surgery (days)	3.52 ± 2.73	3.34 ± 2.58	0.683
Baseline kidney function			
eGFR (MDRD)	89.1 ± 17.6	85.7 ± 19.1	0.146
Serum creatinine (μmol/L)	76.7 ± 19.6	85.2 ± 27.0	0.003
LVEF	62.7 ± 6.9	60.0 ± 8.8	0.006
NYHA III-IV (%)	135 (63.7)	57 (64.8)	0.857
Baseline hemoglobin (g/L)	130.2 ± 17.2	133.2 ± 15.7	0.166
APACHE III	12.6 ± 3.4	13.0 ± 4.0	0.464
EuroSCORE II	1.20 ± 0.71	1.35 ± 0.89	0.161
Surgery type			
CABG (%)	36 (17.1)	10 (11.4)	0.142
Single valve surgery (%)	75 (35.5)	34 (38.6)	0.455
Combined valve surgery (%)	49 (23.3)	22 (25.0)	0.858
CABG + valve surgery (%)	4 (1.9)	3 (3.4)	0.419
Aortic root surgery (%)	47 (22.3)	20 (22.7)	0.941
Intraoperative fluid balance			
Estimated blood loss (ml)	414 ± 313	462 ± 393	0.358
Ultrafiltration (ml)	2295 ± 794	2525 ± 800	0.092
Erythrocytes transfusion (ml)	420 ± 215	470 ± 289	0.144
FFP transfusion (ml)	386 ± 133	531 ± 222	0.005
Surgery variables			
CPB duration (min)	90.1 ± 31.8	108.4 ± 37.1	< 0.001
Cross-clamp duration (min)	55.8 ± 24.9	66.7 ± 25.6	0.004
Dose of vasopressor or inotropes			
Norepinephrine (mg)	6.2 ± 5.7	10.2 ± 13.8	0.003
Epinephrine (mg)	0.4 ± 1.3	1.2 ± 2.6	0.021
Dopamine (mg)	80.6 ± 112.1	86.6 ± 97.2	0.721
Dobutamine (mg)	54.7 ± 72.0	66.1 ± 82.2	0.322
Fluid balance at POD_24h_ (ml)	− 290 ± 1475	− 256 ± 1752	0.876
Fluid infusion (ml)	3804 ± 850	3841 ± 844	0.754
Fluid output (ml)	4157 ± 1520	4325 ± 1514	0.474
Urine output (ml)	2099 ± 721	1789 ± 734	0.002
Outcomes			
CRRT (%)	0 (0.0)	5 (5.7)	0.037
LOS in ICU (hours)	34.8 ± 29.3	47.7 ± 30.8	0.001
LOS in hospital (days)	11.0 ± 2.8	12.1 ± 4.1	0.027
In-hospital mortality (%)	3 (1.4)	5 (5.7)	0.037

The data in the table are expressed as mean ± standard deviation or number (%). Continuous variables were compared using the T test, whereas categorical variables were compared using chi-squared test

### Hemodynamic indices and CSA-AKI

Table 2 shows a comparison of the perioperative hemodynamic parameters in AKI and non-AKI groups. Premorbid baseline BP (including SAP, DAP and MAP) levels were virtually identical in both groups. However, the patients with AKI had greater values of relative ∆MAP (25.3 ± 9.4% vs. 22.5 ± 11.0%; *P =* 0.039), and higher ULR and LLR values of postoperative CVP (10.7 ± 3.5 mmHg vs. 9.8 ± 2.7 mmHg; *P* = 0.013; 5.0 ± 2.2 mmHg vs. 4.5 ± 1.8 mmHg; *P* = 0.048, respectively) in comparison to the non-AKI patients. During the postoperative period, TWA-MAP at 6 h and 12 h was significantly reduced in the AKI groups (all *P* < 0.05). In addition, ULR and LLR values of DPP as well as LLR value of MPP in AKI patients were statistically less than non-AKI patients (51.9 ± 7.1 mmHg vs. 53.7 ± 6.0 mmHg; *P* = 0.027; 46.1 ± 7.1 mmHg vs. 48.4 ± 6.4 mmHg; *P* = 0.008; 65.1 ± 6.8 mmHg vs. 66.7 ± 6.1 mmHg, *P* = 0.037, respectively) (Table 2).

**Table 2:** Perioperative hemodynamic parameters and AKI

	Non-AKI	AKI	*P*-value
Presurgical period			
Baseline SAP (mmHg)	124.6 ± 15.8	127.1 ± 14.1	0.202
Baseline DAP (mmHg)	73.2 ± 9.5	72.1 ± 10.4	0.398
Baseline MAP (mmHg)	90.3 ± 9.6	90.5 ± 9.1	0.894
Surgical period			
TWA-MAP (mmHg)	69.2 ± 7.8	67.7 ± 8.3	0.114
∆MAP relative to baseline (mmHg)	21.0 ± 11.4	23.4 ± 10.0	0.091
%∆ MAP relative to baseline (%)	22.5 ± 11.0	25.3 ± 9.4	0.039
Postsurgical period			
CVP (ULR) (mmHg)	9.8 ± 2.7	10.7 ± 3.5	0.013
CVP (LLR) (mmHg)	4.5 ± 1.8	5.0 ± 2.2	0.048
TWA-SAP (mmHg)	113.3 ± 8.6	113.6 ± 10.5	0.835
TWA-DAP (mmHg)	58.2 ± 6.2	56.8 ± 6.9	0.099
TWA-MAP (mmHg)	76.5 ± 5.8	75.8 ± 6.8	0.313
SPP (ULR) (mmHg)	108.8 ± 8.7	108.6 ± 10.6	0.859
SPP (LLR) (mmHg)	103.5 ± 9.0	102.9 ± 10.4	0.581
DPP (ULR) (mmHg)	53.7 ± 6.0	51.9 ± 7.1	0.027
DPP (LLR) (mmHg)	48.4 ± 6.4	46.1 ± 7.1	0.008
MPP (ULR) (mmHg)	72.1 ± 5.7	70.8 ± 7.0	0.112
MPP (LLR) (mmHg)	66.7 ± 6.1	65.1 ± 6.8	0.037

Continuous variables are expressed as mean ± standard deviation and compared using the T test

### Risk factors for AKI development

Univariate analyses showed that of the perioperative hemodynamic parameters measured, the relative change of ∆MAP, the ULR and LLR values of CVP and DPP, and the LLR value of MPP were implicated in AKI development (all *P* < 0.05) (Table 3). Nevertheless, after adjusting confounding risk factors for the development of CSA-AKI (including age, gender, baseline serum creatinine, baseline LVEF, and CPB duration, fluid balance, PEEP), only the LLR value of DPP was significantly related to the AKI occurrence (odds ratio [OR] for 1 mmHg increase = 0.945; 95% CI 0.894–0.999; *P* = 0.045) (Table 4).

**Table 3:** Univariate analysis with AKI as the outcome variable

Predictor	Odds Ratio	95% CI	*P*-value
Male/Sex (present)	4.504	2.487–8.157	< 0.001
Age (per 1 year increase)	1.030	1.009–1.050	0.004
BMI (per 1 unit increase)	1.109	0.983–1.252	0.092
Hypertension (present)	1.712	1.012–2.896	0.045
LVEF (per 1 unit increase)	0.956	0.926–0.988	0.008
Baseline SCr (per 1 μmol/L increase)	1.017	1.005–1.030	0.005
CPB (per 1 min increase)	1.015	1.007–1.024	0.001
Cross-clamp (per 1 min increase)	1.017	1.005–1.029	0.005
% ∆MAP relative to baseline (per 1 unit increase)	1.026	1.001–1.051	0.040
CVP (ULR) (per 1 mmHg increase)	1.113	1.022–1.211	0.014
CVP (LLR) (per 1 mmHg increase)	1.132	1.000–1.281	0.050
DPP (ULR) (per 1 mmHg increase)	0.956	0.918–0.995	0.028
DPP (LLR) (per 1 mmHg increase)	0.949	0.913–0.987	0.009
MPP (LLR) (per 1 mmHg increase)	0.959	0.921–0.998	0.039

**Table 4:** Multivariable logistic analysis with AKI as the outcome variable

Predictor	Odds Ratio ^a^	95% CI	*P*-value
% ∆MAP relative to baseline (per 1 unit increase)	1.002	0.969–1.037	0.907
CVP (ULR) (per 1 mmHg increase)	1.113	0.997–1.242	0.057
CVP (LLR) (per 1 mmHg increase)	1.160	0.988–1.362	0.071
DPP (ULR) (per 1 mmHg increase)	0.949	0.895–1.007	0.082
DPP (LLR) (per 1 mmHg increase)	0.945	0.894–0.999	0.045
MPP (LLR) (per 1 mmHg increase)	0.951	0.899–1.005	0.076

### Subgroup analyses in patients with chronic hypertension

Subgroup analysis was further carried out in cardiac surgery patients with chronic hypertension (*n* = 91). After adjusting for risk factors for AKI (age, gender, baseline serum creatinine, baseline LVEF, CPB duration, fluid balance, average PEEP), the ULR value of CVP and the LLR value of DPP were both significantly and independently associated with AKI occurrence (OR for 1 mmHg increase of CVP [ULR] = 1.328, 95% CI: 1.070–1.648, *P* = 0.010; OR for 1 mmHg increase of DPP [LLR] = 0.886, 95% CI: 0.793–0.990, *P* = 0.010) (Table 5).

**Table 5:** Subgroup analysis with hypertension: adjusted association with AKI

Predictor	Odds Ratio ^a^	95% CI	*P*-value
% ∆MAP relative to baseline (per 1 unit increase)	0.991	0.924–1.062	0.788
CVP (ULR) (per 1 mmHg increase)	1.328	1.070–1.648	0.010
CVP (LLR) (per 1 mmHg increase)	1.186	0.889–1.583	0.245
DPP (ULR) (per 1 mmHg increase)	0.945	0.856–1.045	0.272
DPP (LLR) (per 1 mmHg increase)	0.886	0.793–0.990	0.033
MPP (LLR) (per 1 mmHg increase)	0.908	0.813–1.014	0.087

## Discussion

Our study revealed that in patients who underwent cardiac surgery, the nadir value of postoperative DPP within 24 h was independently related to the development of AKI. Subgroup analyses further implied that postoperative nadir DPP and peak CVP were both closely linked to AKI development in patients with chronic hypertension.

Several studies have reported that there was no significant association between intraoperative MAP and postoperative AKI [20, 21]. However, the MAP deficit, defined as the difference between the premorbid and intraoperative MAP, was found to be related to the occurrence of AKI [22]. Univariate analysis of our data revealed the relative change of MAP deficit, but not the absolute MAP deficit, was closely linked to the development of CSA-AKI. However, when after adjustment for the risk factors for AKI, there was no relationship between MAP deficit (both relative and absolute) and the occurrence of AKI.

Recently, several studies have demonstrated that MPP or DPP was related to the development or progression of AKI. In a retrospective study of critically ill patients, it was reported that patients with an MPP ≤ 59 mmHg had a higher risk of AKI progression [23]. Moreover, the median value of MPP deficit [=%(baseline MPP-achieved MPP)/baseline MPP] was higher in patients with severe septic AKI when compared to those without this condition [16, 17]. In cardiovascular surgery patients who were vasopressor-dependent, it was demonstrated that deficits in DPP and MPP were significantly related to the progression of AKI [15]. Similarly, we found that postoperative peak and nadir values of DPP and the nadir value of MPP were significantly reduced in patients with AKI. However, after adjustment for confounding factors for AKI, only the nadir value of DPP was independently related to the development of AKI. The difference of DPP between AKI and non-AKI patients in our study was greater than the results from previous studies [15]. Furthermore, given that postoperative MPP levels in our cohort were relatively higher than that in previous studies, no independent associations were detected between postoperative MPP and the development of AKI from our analyses.

Subgroups analyses indicated that the peak value of postoperative CVP also independently contributed to the development of AKI in patients with chronic hypertension. The link between AKI and CVP was most notable in patients with cardiovascular disease [12, 13]. More recently, Williams et al. reported that in cardiovascular surgery patients for CVP increments > 5 mmHg above the 9 mmHg threshold, the risk of developing AKI had an odds ratio of 1.3 [11]. Yang et al. proposed that high CVP value (especially higher than 10 cmH_2_O) at the end of cardiac surgery was independently associated with AKI [24]. Increments of CVP, especially in hypervolemic status, impedes venous return, which results in elevated venous pressure and further decreased cardiac output. Furthermore, an increased venous pressure may trigger an increased renal interstitial pressure, which commonly leads to impaired microcirculatory flow and reduced glomerular filtration rate [25, 26]. Hence, postoperative CVP levels should be closely monitored in patients undergoing cardiac surgery, especially in those who had a history of chronic hypertension.

As far as we know, the present research is the first to have investigated the relationship between perioperative extensive hemodynamic parameters and CSA-AKI. Based on our results, we suggest the nadir level of DPP may serve as a surrogate for indicating the risk of AKI development. In cardiac surgery patients with decreased DPP, excessive fluid resuscitation should be under deliberation in patients with chronic hypertension. Further interventional studies should be carried out to clarify whether potential therapeutic manipulations such as increasing DPP or limiting CVP may prevent AKI development in cardiac surgery patients.

However, our investigation still confers some limitations. First, it was a single-center retrospective study with a relatively small cohort of patients. Our study only enrolled patients who underwent cardiac surgery, limiting the applicability of our findings to general settings. Second, since preoperative CVP values were not available in our database, premorbid perfusion pressure was not taken into account in the analysis. Finally, a causal relationship between relevant hemodynamic variables and the development of AKI may not be established due to the inherent bias of the study design. Therefore, further interventional studies should be carried out to establish possible causal connections between hemodynamic parameters and AKI, which will be beneficial for the hemodynamic management in patients undergoing cardiac surgery.

## Conclusions

Postoperative nadir DPP was independently associated with the development of AKI. Postoperative peak CVP was significant for AKI in patients with chronic hypertension. Further investigations are needed in the future to verify whether advanced hemodynamic management (specifically targeting DPP and CVP) may reduce the incidence of CSA-AKI.

## Data Availability

The datasets used and/or analyzed during the current study are available from the corresponding author on reasonable request.

## Abbreviations

AKI: Acute kidney injury
APACHE: Acute Physiology and Chronic Health Evaluation
BP: Blood pressure
CI: Confidence interval
CPB: Cardiopulmonary bypass
CSA-AKI: Cardiac surgery-associated acute kidney injury
CVP: Central venous pressure
DAP: Diastolic arterial pressure
DPP: Diastolic perfusion pressure
eGFR: Estimated glomerular filtration rate
ICU: Intensive care unit
LLR: Lower limit of the range
LVEF: Left ventricular ejection fraction
MAP: Mean arterial pressure
MDRD: Modification of Diet in Renal Disease
MPP: Mean perfusion pressure
OR: Odds ratio
PEEP: Positive end-expiratory pressure
SAP: Systolic arterial pressure
SPP: Systolic perfusion pressure
TWA: Time-weighted average
ULR: Upper limit of the range
